# Colorimetric Gas Sensing Washable Threads for Smart Textiles

**DOI:** 10.1038/s41598-019-42054-8

**Published:** 2019-04-04

**Authors:** Rachel E. Owyeung, Matthew J. Panzer, Sameer R. Sonkusale

**Affiliations:** 1 Department of Chemical and Biological Engineering, Tufts University Science and Technology Center, 4 Colby Street, Medford, MA 02155 USA; 2 Department of Electrical and Computer Engineering, Tufts University Halligan Hall, 161 College Ave, Medford, MA 02155 USA; 3 Nano Lab, Tufts University Advanced Technology Laboratory, 200 Boston Suite 2600, Medford, MA 02155 USA

## Abstract

A fabrication method for a stable entrapment of optically responsive dyes on a thread substrate is proposed to move towards a detection system that can be integrated into clothing. We use the dyes 5,10,15,20-Tetraphenyl-21H,23H-porphine manganese(III) chloride (MnTPP), methyl red (MR), and bromothymol blue (BTB), for a proof-of-concept. Our optical approach utilizes a smartphone to extract and track changes in the red (R), green (G) and blue (B) channel of the acquired images of the thread to detect the presence of an analyte. We demonstrate sensing of 50–1000 ppm of vapors of ammonia and hydrogen chloride, components commonly found in cleaning supplies, fertilizer, and the production of materials, as well as dissolved gas sensing of ammonia. The devices are shown to be stable over time and with agitation in a centrifuge. This is attributed to the unique dual step fabrication process that entraps the dye in a stable manner. The facile fabrication of colorimetric gas sensing washable threads is ideal for the next generation of smart textile and intelligent clothing.

## Introduction

The detection of volatile gases in the environment is essential for applications in human health^1^, monitoring food spoilage or allergens^2^, and assessing public and workplace safety^3^. Popular techniques for gas sensing include electrochemical detection of oxidation of target analyte, optical detection of chemically responsive materials, and detection of light induced ionization of gases using photo-ionized detectors (PIDs)^4,5^. These techniques have achieved high sensitivity and selectivity, though the vast range of applications (e.g. portable, wearable) prove a need for further operational advancements. These include the ability to distinguish between many analytes detected in presence of complex air background, while enabling equipment-free read out, training-free usage, and facile fabrication approaches to produce robust and reusable sensors. This will improve the affordability and ease of use to grant low resource communities access to these necessary technologies.

In this regard, optical detection offers advantages over other sensing techniques, as scanners and smartphones can image and analyze the color difference due to sensing instance^2,6^. The advancements in cell phone camera quality and accessibility also negate the need to incorporate lighting and lenses within the same device. Suslick and co-workers have made substantial findings in this field, regarding the development of an optoelectronic nose. Their group used arrays of chemically responsive dyes to sense and distinguish between volatile organic compounds, beer, and explosives, to name a few, for a wide field of applications^4,7–10^. Their prior work focused on low-cost, but one time use optical gas sensors on paper^4,10^.

In this optoelectronic nose, utilizing an array of dyes mimics the biological olfactory systems, where the specificity in odor detection originates from pattern recognition of responses of many cross-reactive olfactory receptors^4,8^. Types of dyes that are commonly used in these optoelectronic nose arrays include Bronsted acidic or basic dyes, Lewis acidic or basic dyes, redox responsive dyes, or dyes with large permanent dipoles including solvatochromic or zwitterionic dyes^4^. The incorporation of several dyes within each category leads to sensor diversity^10,11^, with the ensemble response able to distinguish between different analytes detected.

Threads have become popular substrates for low-cost diagnostic platforms and wearable technology advances. Thread-based e-textiles have been employed in various health care monitoring, such as electrocardiograms, electromyograms, and other mechanical signals^12–15^. Like paper-based approaches, threads are flexible and have favorable wicking properties^16^. Previously, our group has demonstrated paper-based approaches for environmental sensing studies^2,11^. Contrastingly, threads offer a three-dimensional area for analytical measurements and have an added benefit of easier integration into smart clothing. As such, our group developed a toolkit of chemical and electrical sensing techniques using thread substrates^15–20^. Threads are especially suitable for sensing applications, as their geometry favors a high surface area to volume ratio for achieving high sensitivity. Threads have been employed for gas sensing using conductivity changes^21,22^. Seesaard *et al*. demonstrated gas sensing of a few common volatile compounds, ammonium hydroxide, ethanol and pyridine to name a few^23^. However, little attention in literature has been dedicated to the switch from paper-based substrates to threads for optical sensing techniques. In one of the first studies to do so, Galpothdeniya *et al*. utilized an ion exchange reaction to pair anionic pH indicator dyes with an ionic liquid cation to immobilize the dye without the use of a matrix^24^. By doing so, they achieved a pH sensing platform on both filter paper and cotton thread substrates for the detection of aqueous solutions. This method showed stability in aqueous solutions, but was limited to anionic dyes to complex with the ionic liquid.

**Figure 1:**
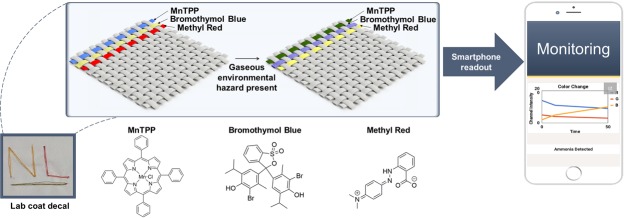
Concept of the gas sensing washable threads. Illustrates the thread-based sensors integrated into a textile patch. As the gas passes over the patch, the optical dye changes conformation, resulting in a color change. Color change is determined via smartphone readout.

We propose a fabrication method for an optical sensing platform on thread to extend sensor diversity beyond anionic dyes. This proof-of-concept design can utilize a wide range of dye categories to mimic the olfactory system and provide distinction between what could be many types of analytes sensed. In this work, an acidic and basic pH indicator, as well as a metalloporphyrin dye was chosen for demonstration of trapping different dye types via our proposed entrapment method. Importantly, this method focuses on a stable physical entrapment of the dyes to retain the washability and flexibility of the original thread substrates. This dual step technique involves first an acetic acid cleaning of thread fibers to enable more dye penetration, followed by a hydrophobic silicone coating. Rigorous validation using repeated washing demonstrates reusable environmental sensing on a clothing platform which is advantageous towards the next generation of wearable environmental sensors conceptually demonstrated in Fig. 1.

## Results

### Fabrication of the stable thread-based devices

**Figure 2:**
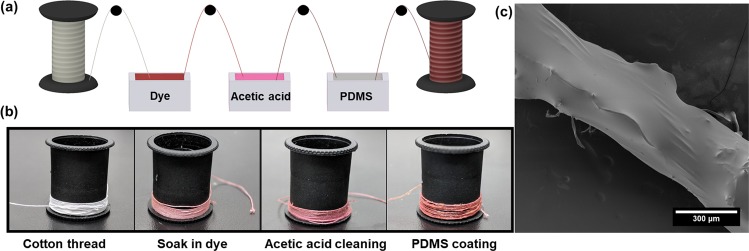
Fabrication scheme of the gas sensing washable threads. (**a**) Device fabrication procedure for thread coating, (**b**) resulting threads, and (**c**) SEM image of the final thread device.

**Figure 3:**
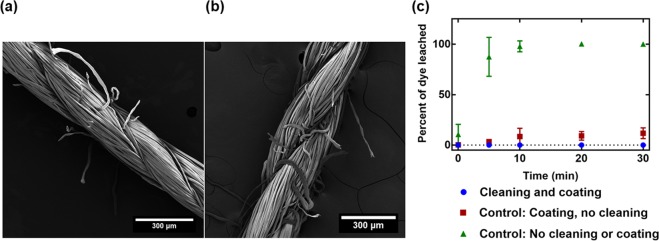
Effect of acetic acid cleaning on thread washability. SEM image results of the (**a**) bare cotton thread without treatment 224 ± 11 μm, (**b**) bare cotton thread after acetic acid treatment 290 ± 26 μm. (**c**) Effects of PDMS coating and acetic acid cleaning on the stability of the sensors in an aqueous environment with time.

We achieve stable entrapment of several optical dye types through a simple fabrication method, illustrated in Fig. 2. This facile process involves three dip and dry steps. The process begins by soaking the original thread material in a given dye solution, where the thread then proceeds to two treatment methods for stability. The first of these treatments is an acetic acid cleaning of the threads. Acetic acid has been used to roughen the surface of fibers by removing some of the non-cellulose components^25^. Here, we believe the choice of acetic acid has a twofold effect on suppressing the leaching of dyes. First, it partially opens the thread fibers, possibly by removing any wax or non-cellulosic coating, allowing for more attractive interactions between the dye molecules and the cotton thread. Indeed, SEM results confirm an increase in thread diameter upon soaking in acetic acid, from 224 to 290 μm (averaged between three points on three samples) as shown in Fig. 3(a,b). Second, it washes away excess dye not properly adhered to the cotton surface. The acetic acid cleaning is followed by a coating of polydimethylsiloxane (PDMS) for a physical entrapment of the dyes. The coating is realized by pulling the thread through the pre-cured PDMS mixture. PDMS was chosen for its elastomeric and hydrophobic properties, allowing the devices to remain flexible and conformable, similar to that of the original cotton thread substrates and to better repel aqueous solutions during washing. Additionally, PDMS is known to be gas permeable, such that the target analyte can still reach the optical dyes^26–30^. The PDMS coating allows for the thread to withstand aqueous conditions that would normally wash away a significant portion of the dye that was adhered onto the original thread substrate. Figure 3(c) highlights the effectiveness of the dual step fabrication as it provides stable entrapment of bromothymol blue (BTB) on a cotton thread, showing little to no dye leached with time. Adding a coating of PDMS without acetic acid cleaning (control) shows a stable entrapment, but still some dye leaching (around 10–20%). Dyed threads without acetic acid cleaning and without PDMS coating (control) show significant leaching, up to 100% over 30 minutes. As such, we can see that this acetic acid cleaning, then PDMS coating fabrication scheme leads to stably trapped dyes in aqueous solutions.

### Stability of the as-fabricated sensors

**Figure 4:**
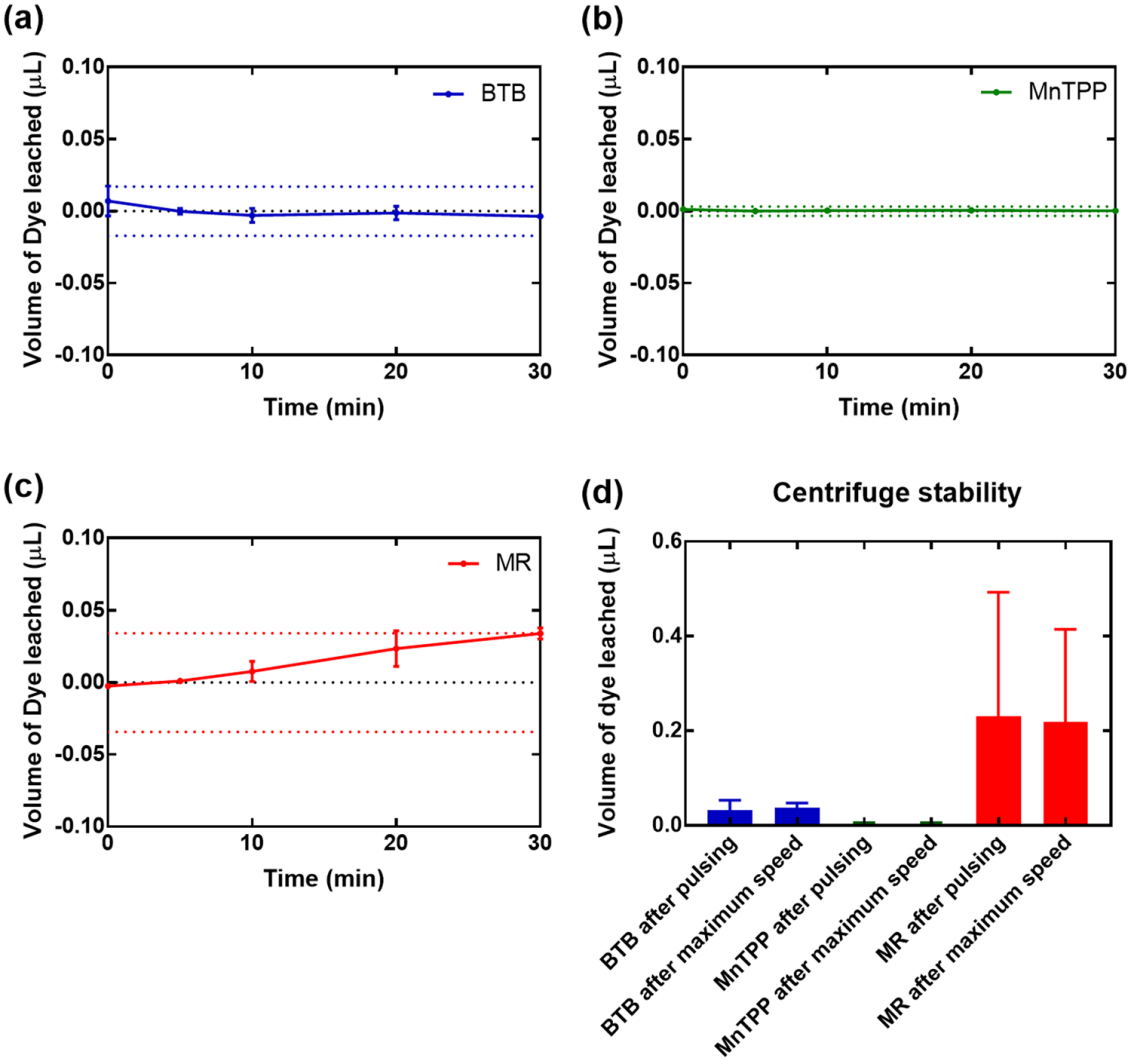
Stability results of the three different sensor types. (**a**–**c**) Submersion in aqueous solution of the bromothymol blue (BTB), 5,10,15,20-Tetraphenyl-21H,23H-porphine manganese(III) chloride (MnTPP), and methyl red (MR) gas sensing threads, respectively. (**d**) Agitation of the three different sensor types through pulsing or spinning at maximum speed to simulate washing environments.

To view stability, we performed two tests for each dye type, to test longevity and agitation effects, each in triplicate. For the longevity tests, we soaked the devices in water, and then performed UV-VIS absorption scans on the water after removing the device at known time periods. The results are shown in Fig. 4(a–c) for BTB, MnTPP, and MR, respectively. The dotted lines in each plot indicate the limit of detection of the instrument converted to volume of dye for each particular dye type. We can see no values above the limit of detection of the UV-VIS system in bromothymol blue (BTB) and 5,10,15,20-Tetraphenyl-21H,23H-porphine manganese(III) chloride (MnTPP) devices, indicating negligible leaching over a 30-minute period. For methyl red (MR), we do see a slight increasing trend over the 30 minutes, but these values are still within the limit of detection of the UV-VIS.

To replicate agitation forces experienced when washing, we performed several tests using a centrifuge, shown in Fig. 4(d). The pulsing experiments were used to demonstrate effects of acceleration and deceleration. Then, the devices were held at 6400 rpm. Again, BTB and MnTPP showed little to no leaching, and MR showed some leaching but retained over 90% of the original dye content. These results are promising, as they illustrate that the coating method retains a majority of the sensing agent on the thread during significant agitation. Both sets of stability data are for three separate sets of fabricated sensors for each of the three dye types used for fabrication validation here. Of course, the as-assembled devices with improved fabrication methods could show more reproducible results.

### Gas sensing demonstration

**Figure 5:**
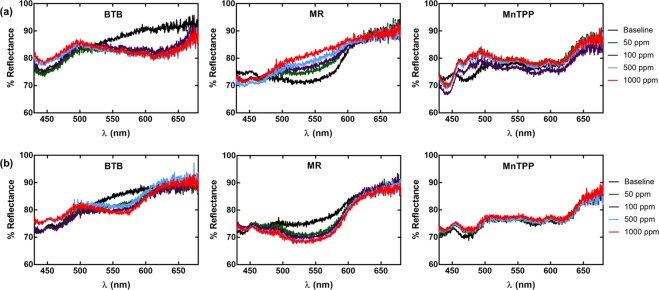
Gas sensing demonstration – Spectroscopy measurements. Spectra of each thread device upon exposure to different concentrations of (**a**) ammonia or (**b**) HCl.

As proof of concept, we demonstrated sensing of two common VOCs, ammonia and HCl gas using spectroscopy techniques for initial validation and later, comparing optical images of the thread devices. These two gases were chosen to highlight the sensing of a base with lone pair electrons (ammonia) and a Bronsted acid (HCl), as they should interact with the dyes chosen for our proof of concept system. BTB is a weak acid. The deprotonation of a hydroxyl group begins a rearrangement of the conjugated system and the formation of a sulfonate (SO_3_^−^) group^31^. The resulting arrangement appears blue. Likewise, this molecule can be protonated to reverse the rearrangement and appears yellow. MR, similarly is a weak acid, and also changes color due to a protonation or deprotonation event. For this molecule, protonation causes a rearrangement of the conjugated molecule, shifting the diimide group to a hydrozone structure. The protonation event shifts MR’s appearance from yellow to red. MnTPP is a different class of dye. Instead of a protonation or deprotonation event causing a conformation change, the presence or absence of a Lewis base pair of electrons causes a rearrangement of this metalloporphyrin^32,33^. For quantitative validation of the optical sensors, we first take reflectance measurements of the three different thread devices of different dye types and test their spectral response to ammonia and HCl gas. A detailed description and image of the reflectance measurement setup can be found in Supplementary information and Supplementary Fig. S3. The results are shown in Fig. 5(a,b) (ammonia and HCl, respectively). From these figures, we can see that some devices show changes with respect to a few different wavelengths as concentration increases. For example, BTB exhibits reflectance intensity changes around 440 and 610 nm for increasing concentrations of ammonia and 440 and 570 nm for HCl. MR exhibits a reflectance intensity increase around 550 nm for ammonia, and a decrease in intensity around 550 nm for HCl. For MnTPP, we can observe a decrease in a peak around 472 nm and an emergence in two peaks around 467 nm and 485 nm. Also, we do not observe a noticeable trend for MnTPP in the presence of HCl, which is expected, as HCl does not have lone pair electrons for MnTPP to interact with. Some nonlinearities exist, though this could be due to changes in scattering events upon moving the sample to test, as a change in surface area of threads in the path of the light will affect the scattering. This was minimized by using an integrating sphere but should be noted.

**Figure 6:**
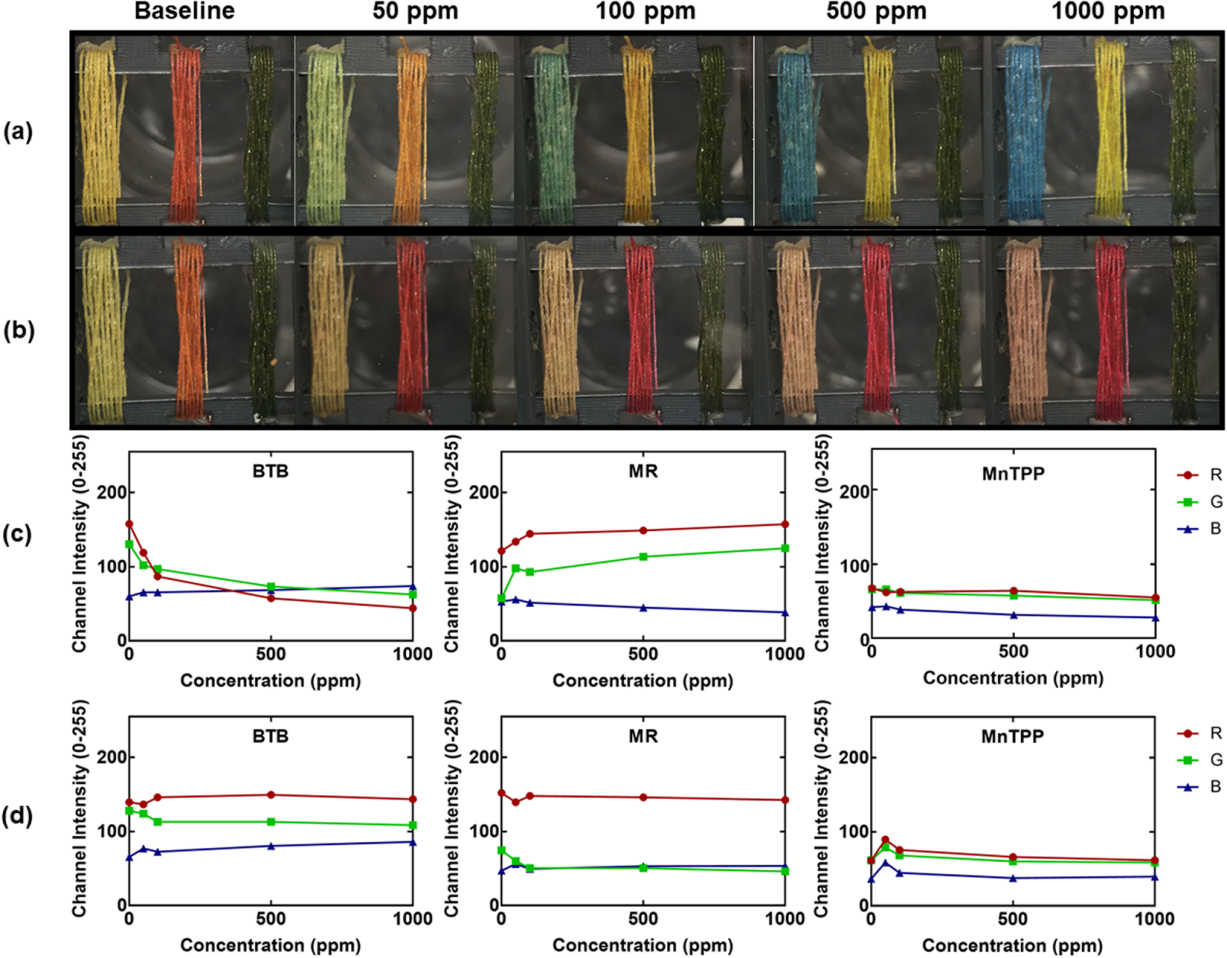
Gas sensing demonstration – Optical Images. Optical images of BTB, MR, and MnTPP devices for different concentrations of (**a**) ammonia or (**b**) HCl and the corresponding RGB color information extracted from the optical images for (**c**) ammonia and (**d**) HCl.

For this reason, as well as the cost barriers of spectrometers and optical setups, we also report color change information via optical images before and after exposure to different concentrations of these gases. The results are shown with Fig. 6(a,b) optical images of the devices at different concentrations and Fig. 6(c,d) the corresponding extracted RGB color information using the smartphone readout. From the optical images, there is a clear difference in the threads for both gases, most notably in the BTB and MR threads, which agrees with the spectroscopy data shown in Fig. 5(a,b). For example, upon exposure to ammonia, the BTB thread shifts from yellow to blue. From the extracted color information, we can see from Fig. 6(c) a significant decrease in channel intensity of the red and green components, and an increase in blue component upon increased concentration levels of ammonia. Though some of these results seem like minor intensity changes, the culmination of all three channels together generates a unique color, which can be rather different from each other upon single digit shifts in intensity of each component. We can see this phenomenon clearly when comparing the optical image changes shown in Fig. 6(a,b) with the extracted color information in Fig. 6(c,d). For this simple proof of concept, smartphone readout is not necessary to see the changes of the sensors. However, for more robust and complex color signatures, smartphone readout becomes increasingly important for pattern recognition and is therefore included to demonstrate for a more robust and complex device. Ammonia and HCl were chosen to demonstrate sensing from the three different types of dyes used to functionalize the thread devices, though expansion of the test gases could lead to greater insights in the discriminative power of an array of these thread-based sensors.

Regarding the time sensitivity of the sensors, the thickness of the PDMS greatly effects the response time. To examine this further, we monitored the R channel intensity of the BTB device upon exposure to 100 ppm ammonia with time in triplicate for three different coating thicknesses. Results can be found in Supplementary Fig. 2 and are discussed there. Briefly, PDMS thickness effects the rapidness of the color change, as expected. Yet, there is a trade-off for stability versus response time for these wearable thread-based sensors. Optimizing the fabrication process could improve upon this response time for future implementations.

**Figure 7:**
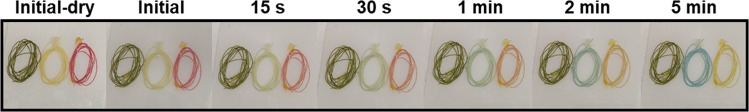
Dissolved gas sensing demonstration. Washable gas sensor responses upon exposure to dissolved ammonia gas in an aqueous environment at different time periods over a span of 5 minutes. Sensor dye types correspond to MnTPP, BTB, and MR, from left to right.

Finally, we highlight the versatility of washable gas sensors through a secondary application. We exploit the ability of the gas sensors to sustain exposure to an aqueous environment, such that it can also be used for dissolved gas sensing applications. These applications include monitoring exposure to hazardous spills in laboratory environment, or greenhouse gas emissions from ocean environments and ecosystems as well as the overall health of aquatic ecosystems^34,35^. Here, we monitor how the washable gas sensors behave upon exposure to dissolved ammonia. Ammonia runoff from use in fertilizers or sewage interfering with marine life is quite common^36^. We bubbled ammonia gas into DI water for 1 minute and monitored how the sensors changed over the span of 5 minutes. The results from this can be seen in Fig. 7. There is a noticable change in the sensors that resembles the color change shown for the ammonia sensing in Fig. 6(a). Notably, no leaching of dyes is observed, again validating the washability of these thread-based gas sensors. These washable gas sensors as dissolved gas sensors could be extended to sense other dissolved gases such as CO_2_ for example^37^ or VOCs during oil and gas exploration^38^.

## Discussion

A simple dual step fabrication process with a dip and dry approach has been presented that allows for the entrapment of multiple dye types on thread substrates for use in wearable textiles applications. A unique method of acetic acid cleaning followed by physical entrapment via PDMS coating allows for thread-based optical sensors to withstand washing conditions. Notably, this method is not limited to a specific dye type, as we showed acidic, basic, and metalloporphyrin dye examples trapped via this method. The thread sensors retain their functionality without leaching as demonstrated for VOC sensing even during dissolved gas testing in aqueous environments. Though verified by reflectance meassurements via spectrometer, smart phone readout offers an instrument-free detection of color change from these colorimetric threads if more robust, complex color signatures need to be detected than can be easily perceived by the human eye. The platform can be scaled up by using different chemoresponsive dyes in each thread for sensor diversity to expand the distinguishing power between many analytes. The ability to use these sensors as washable gas sensors integrated into textiles or as dissolved gas sensors further expands the potential applications for these devices. These colorimetric threads can form the basis of the next generation of smart textiles for intimate environmental monitoring of various volatile gases.

## Methods

### Preparation of dye solutions

Methyl red, bromothymol blue, and 5,10,15,20-Tetraphenyl-21H,23H-porphine manganese(III) chloride dyes (Sigma Aldrich) were prepared in a 0.5 (w/v)% solution of ethanol. Each dye solution was sonicated for 5 minutes (Sharpertek Stamina XP) or until fully dissolved. Before use, dyes were vortexed (Fisher Scientific Digital Vortex) for 1 minute to ensure homogenous solution.

### Coating of thread substrate

Commercially purchased cotton threads (Coats Cotton All-purpose) were soaked in a dye solution for 10 minutes. After, the threads are moved to a solution of 8(v/v)% acetic acid for 10 minutes. Upon air drying, the functionalized threads are drawn through PDMS (Sylgard 10:1 base to elastomer) and are cured with heat (60 °C) for at least 2 hours or until fully cured. This process is repeated to generate individual threads for each type of dye used in the sensor array. For thickness comparisons, PDMS thicknesses were varied through multiple coatings.

### Gas testing

All gas testing was performed in a custom acrylic chamber laser cut to size via CO_2_ cutter (Boss LS-1416). A custom holder was 3D printed (Voxel8, Somerville, MA USA) to hold the sensors during measurement at the top of the chamber. The analyte, of ammonium hydroxide or Hydrochloric acid 38% (Sigma Aldrich), was placed at the bottom of the chamber and allowed to diffuse into the air within the chamber to reach the sensor. Humidity levels did not surpass 10% for any concentration tested (Pro’sKit USA, NT-312). For dissolved gas testing, gaseous ammonia was bubbled into DI water via 250 mL capacity bubbler (ACE Glass Incorporated, USA) for 1 minute and then was poured over sensors.

### Spectroscopy measurements

Experimental setup for spectroscopy is explained in more detail in Supplementary information. Briefly, light from an LED driver (Mightex Systems, USA) passes through the thread device held by a custom built PMMA holder, where the light is diffused in an integrating sphere (Newport, USA). The detector is a Flame Spectrometer (Ocean Optics, USA) and records reflectance spectra via Oceanview software (Version 1.6.3, Ocean Optics, USA).

### RGB information gathering

For color quantification, the RGB color model was used to store the color information of a sensor as three integer values, red (R), green (G), and blue (B). The RGB information was gathered by imaging the sensor before and after exposure to an analyte via an iPhone 6 smartphone camera (Apple). Images were loaded into a custom MATLAB program (Version 2016b) to extract average R, G, and B channel color information for the cropped sensor area. Notably, these averages were normalized to a known color reference to account for differences in background lighting quality between measurements.

### Washing tests

Calibration curves for each individual dye used were created via UV-VIS spectroscopy (Evolution 220 Thermo Fisher). A known amount of DI water was loaded into a quartz cuvette (Suprasil, Fisher Scientific) and a microliter of dye was added at a time to generate an absorbance response curve for a given concentration of dye. This curve was used to approximate the amounts of dye leached for both stability versus time and centrifuge stability data. For stability versus time, the device was submerged into the solution of the cuvette and left to soak. At indicated time periods, an absorbance scan was performed on the solution in the cuvette. During a measurement, the device was pushed to the side of the cuvette to not obstruct laser path and to avoid losing any dye from being seen by the absorbance measurements. For centrifuge stability measurements, the device was placed in an Eppendorf tube and was either pulsed in a centrifuge (Qualitron Inc., DW-41–115) from 0 to 6400 rpm 10 times or spun at maximum speed 6400 rpm for 30 s depending on the test.

### Scanning Electron Microscopy (SEM)

SEM images were taken on a Tescan VEGA3 with an acceleration voltage of 10 kV and beam intensity of 10. Prior to imaging, the samples were coated with a layer of 10 nm Au via sputter deposition.

## Supplementary information

Supplementary Information
