# Supplementary material for: Colorimetric Gas Sensing Washable Threads for Smart Textiles
Source: Sci Rep. 2019 Apr 4;9:5607. doi: 10.1038/s41598-019-42054-8 (PMC6449334; doi:10.1038/s41598-019-42054-8)
Supplement: Supplementary file 1 — Supplementary Information [file 41598_2019_42054_MOESM1_ESM.docx]

Supplementary Information

Colorimetric Gas Sensing Washable Threads for Smart Textiles

Rachel E. Owyeung^1,3^, Matthew J. Panzer^1^, Sameer R. Sonkusale^2,3,^*

^1^Department of Chemical and Biological Engineering, Tufts University

Science and Technology Center, 4 Colby Street, Medford MA 02155

^2^Department of Electrical and Computer Engineering, Tufts University

Halligan Hall, 161 College Ave, Medford MA 02155

^3^Nano Lab, Tufts University

Advanced Technology Laboratory, 200 Boston Suite 2600, Medford MA 02155

Corresponding author. Tel.: +1 6176275113; fax: +1 6176273220. E-mail address: sameer@ece.tufts.edu (S.R. Sonkusale). Address: 200 Boston Suite 2600, Medford MA 02155

**
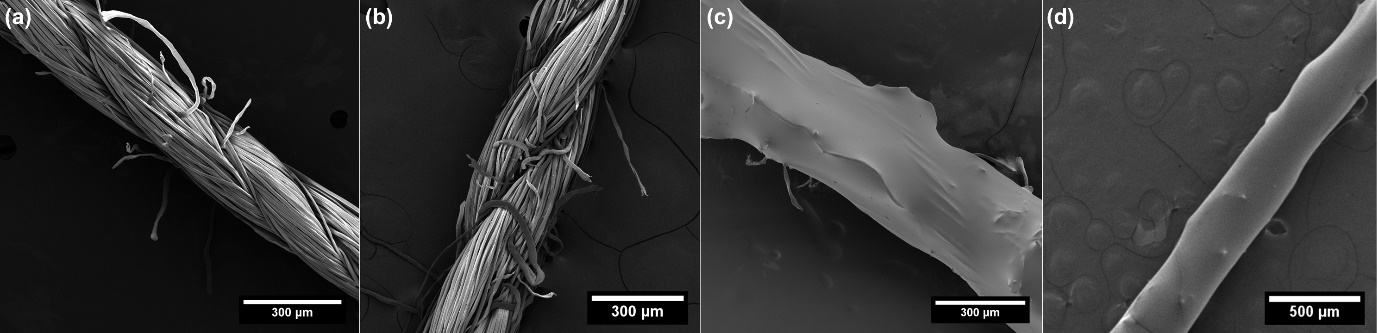
**

**Supplementary Fig. S1.** SEM image results of the a) bare cotton thread without treatment $224\pm11 \mu m$, b) bare cotton thread after acetic acid treatment$290\pm26 \mu m$, c) PDMS coated cotton thread of thickness T1,$390\pm42 \mu m$ and d) PDMS coated cotton thread of thickness T2, 463$\pm42 \mu m$.

**
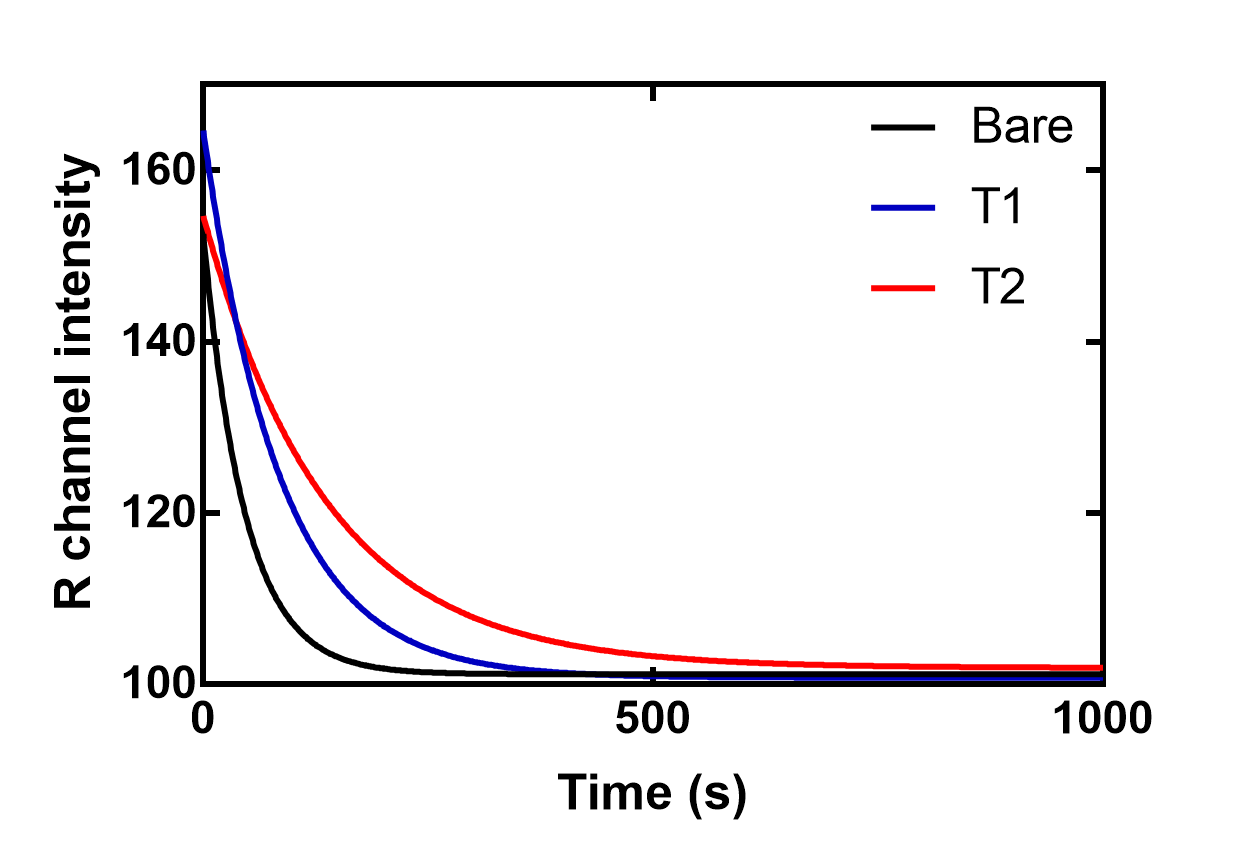
**

**Supplementary Fig. 2.** Thickness dependence of PDMS membrane on BTB R channel intensity response with time. Bare: bare cotton thread after acetic acid treatment$290\pm26 \mu m$, t1: PDMS coated cotton thread of thickness T1,$390\pm42 \mu m$ and t2: PDMS coated cotton thread of thickness T2, 463$\pm42 \mu m$ The test was performed using 100 ppm ammonia exposure.

**PDMS thickness.** “Bare” indicates no coating of PDMS (yet still an acetic acid wash step), T1 indicates a thickness of $390\pm42 \mu m$ and T2 indicates a thickness of 463$\pm42 \mu m$ (same as in Supplementary Fig. S1). As expected, Supplementary Fig. S2 shows PDMS thickness effects the rapidness of the color change. T1 is the thickness used in all other experiments, though one would expect a tradeoff between time sensitivity and leaching stability of the dyes.

Increased thickness of the surrounding PDMS coating hinders the speed at which the optical dyes change conformation to elicit a color change. Interestingly, the three thicknesses had different initial and final channel values. This is likely due to the PDMS membrane altering the apparent color due to light scattering. Using a steady channel intensity of 101, the bare thread half-life was 34.8$\pm$13s, while the half-life of T1 was 70.2$\pm$11.5s and the half-life of T2 was 101.5$\pm$36.2s. Here, half-life indicates the time for the thread’s color intensity to decrease by half, thus a shorter half-life indicates a faster response. Notably, the PDMS coating shown for these experiments doubled the half-life compared to the bare thread, and an even thicker coating nearly tripled the original half-life response.


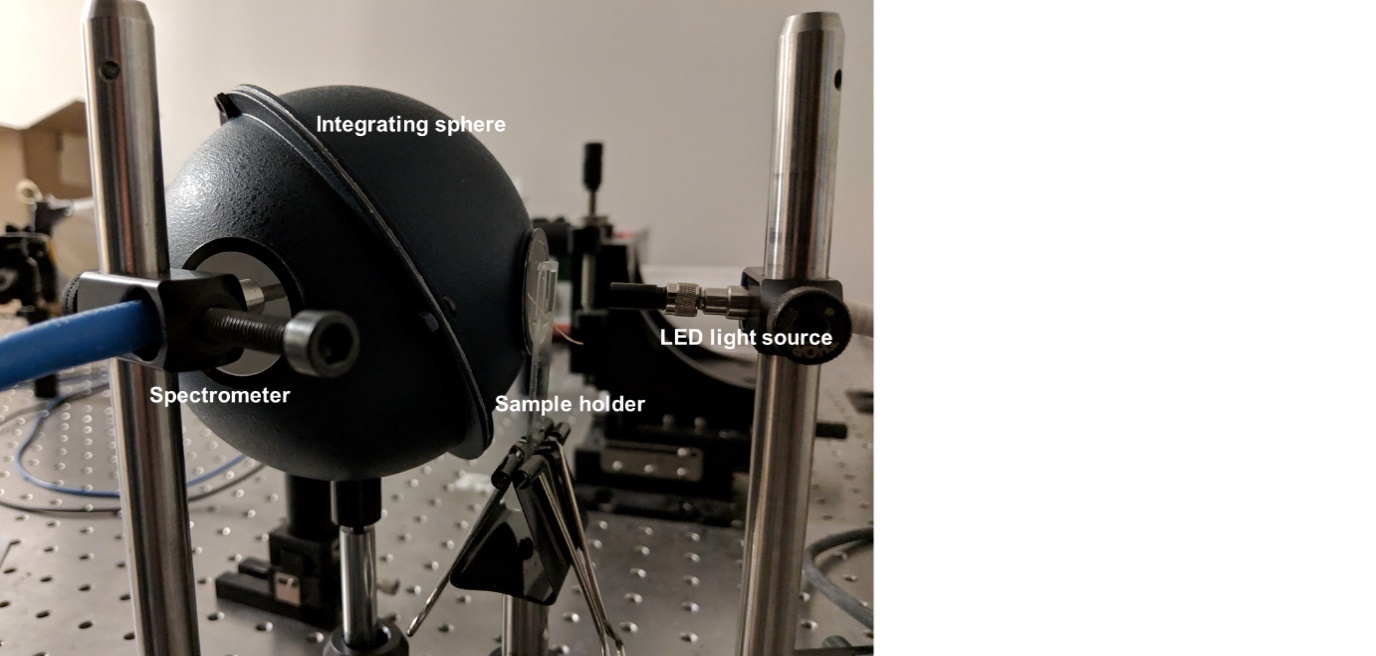


**Supplementary Fig. S3.** Image of the reflectance measurements setup.

**Spectroscopy setup.** Fig. S3 shows the optical setup for reflectance measurements of the thread devices. Light from an LED source passes through the thread sample that is held inside the integrating sphere using a custom PMMA sample holder. This ensures that the integrating sphere is able to capture and diffuse much of the scattered light from the thread, but does not allow the thread to touch the reflective coating inside, as this would damage the integrating sphere. The spectrometer takes data from the 90 degree port, as shown. An integrating sphere was needed to perform spectroscopy measurements of the thread optical sensors, which uniformly diffuses light. Without the integrating sphere, there is very little light that reaches the detector, as most of the light that interacts with the matter (in this case, the threads) is scattered. Also, it should be noted that color measurements depend heavily on the light source that interacts with the matter, as it is the reflectance from that light source that we perceive as color. Therefore, a sample that is illuminated by an LED will appear to have a different color than one illuminated by a fluorescent light, for example. This can explain why the color information from the spectrometer differs from the optical images taken from the smartphone.


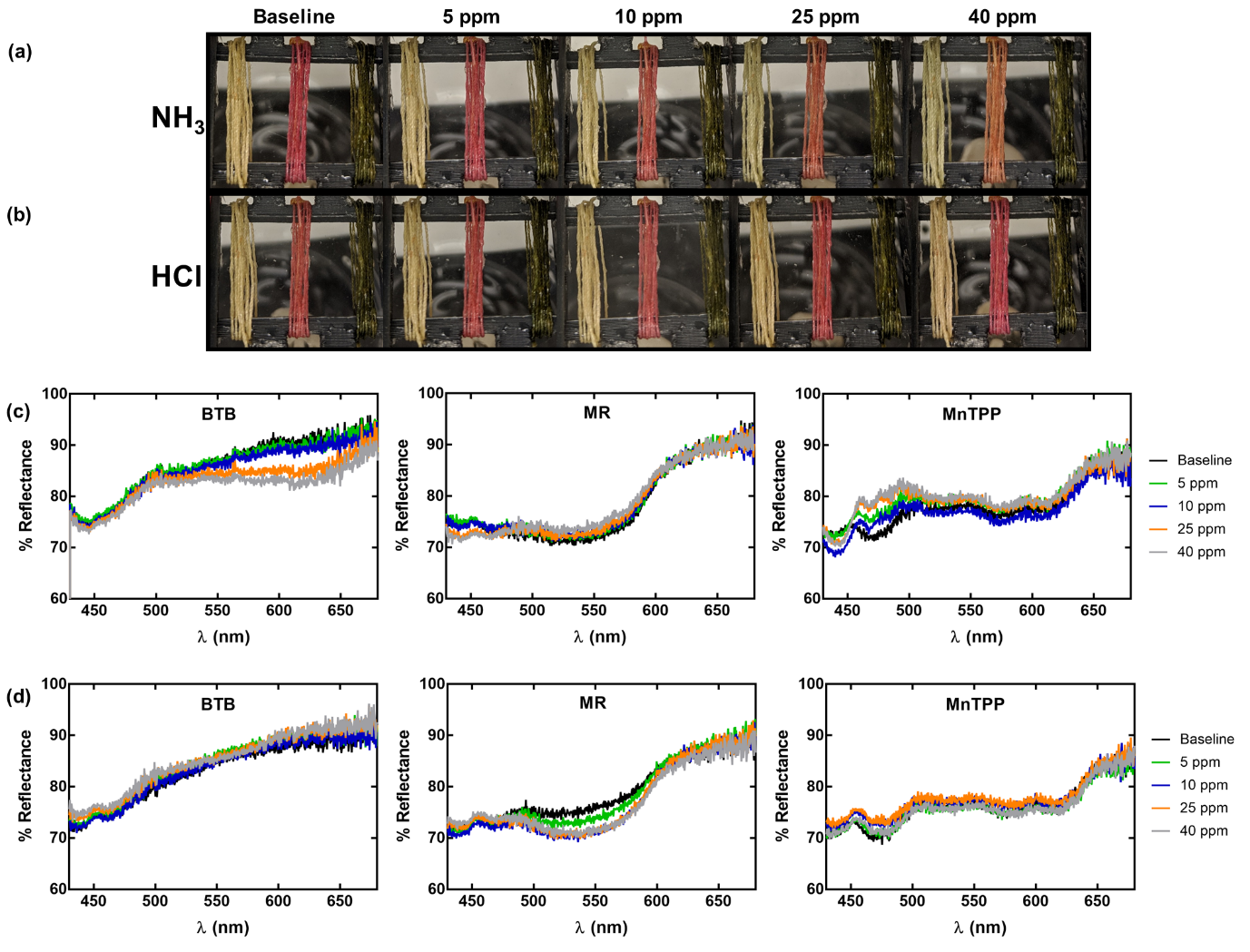


**Supplementary Fig. S4.** Color changing information via optical images of thread devices (BTB, MR, and MnTPP from left to right) at lower concentrations of (a) ammonia and (b) HCl and reflectance data of thread devices at lower concentrations of (c) ammonia and (d) HCl.

**Low concentration testing.** To determine an approximate detection limit for each technique for determining color change, low concentrations of each gas were used to see when a noticable change was present. As expected, there is a difference in perceptive change for the two techniques and for the different dye molecules. For example, the optical images show MR changing from bright red to orange around 10 ppm ammonia gas (Fig. S4(a)) that is not as intuitive to see from Fig. S4(c). Conversely, the reflectance measurements show an apparent change as low as 5 ppm HCl gas for the MR dyes (Fig. S4(d)), that is not as intuitive to see from Fig. S4(b). It should be noted that all threads tested were 32 cm long, and that these limits are effective limits based on the technique and length of thread.
